# Effect of heterogeneous investment induced by payoff and emotion on cooperation in public goods games by considering memory decline effects

**DOI:** 10.1371/journal.pone.0281648

**Published:** 2023-02-10

**Authors:** Hui Long, Rizhao Gong, Jiaqian Yao, Qian Li

**Affiliations:** School of Business, Hunan University of Science and Technology, Xiangtan, China; Zhejiang University of Finance and Economics, CHINA

## Abstract

Payoff, emotion, and historical memory directly determine investment decision-making for incomplete rational men in a public goods game (PGG). How these factors affect investment and cooperation behavior has not been investigated yet. Thus, we proposed a new investment model involving theses three factors to examine its coupling effect on cooperation in PGG. An emotional increment was employed to describe the emotional change in every round by supposing an investor’ pleasure to a cooperator but regret to a defector. Furthermore, an emotional index was formed by accumulating these historical changes with a memory decline effect. Then an investment formula was proposed by considering this emotional index and a historical payoff. Moreover, the cooperation level affected by these factors was investigated. Results show a mutually reinforcing relationship between emotional and payoff investments. A poor memory capacity coefficient allows defectors to change their behaviors but produces some opportunists. A large memory length results in a high cooperator fraction but is not suggested to be too large.

## Introduction

Cooperation is typical behavior in human life and the animal world. Cooperative behavior means that a co-operator benefits other by paying some cost and that a defector gets a payoff without effort. In theory, no one prefers to take cooperative behavior under rational and natural selection. However, this is not true in real life because commonplace cooperation exists in energy development, environmental protection issues, supply chains among companies, et cetera. How to explain these cooperation behaviors in real life has become a hot topic and has attracted much attention worldwide. As the public goods game (PGG) is a helpful tool in explaining the evolution of cooperation behaviors, it is widely employed in national defense, public transport, and in environmental protection issues, et al. [1–5]. Regarding existing reports about a PGG, most researchers mainly focused on a homogeneous or a simple heterogeneous investment caused by some rational factors [6–9]. These investigations have a common shortcoming in explaining the complex cooperation behaviors among incomplete rational men because the investments for incomplete rational men are usually affected by some rational or emotional factors, and so forth.

Rational investments have been widely explored for many years. Many researchers proposed rational investment correlations by considering the degree of a focal player, the groups’ quality, the reputation, the payoff, et cetera. For the investment induced by the degree, a player assigns the investment to a focal player according to the degree of this focal player. This heterogeneous investment is a fixed investment because the investment is determined by the network structure and keeps unchanged from the second round [10–14]. For the investment by the group quality, its investment is allocated according to the fraction of cooperators, implying that most investments are contributed to high-quality groups. This investment is a benefit for improving the cooperation level in a system but is unfair to some cooperators in a low-quality group [15–20]. The investment induced by reputation is determined by the total reputation of players in the group, which means that cooperators prefer to invest more in a group with a higher reputation [21–23]. This investment strategy forces individuals with a poor reputation to adopt a cooperation strategy to improve their reputation. For the investment induced by the payoff, a cooperator contributes to a group according to his previous payoff obtained from this group. This investment method can dynamically update its investment value and is characterized by solid adaptability [24–31]. Above these rational investments, researchers employed the one induced by payoff most widely in true life because it effectively explains a common and fair phenomenon that a cooperator invests more in the group from which he gets more and less to the group from which he gets less.

Except for rational investment, emotion also affects the investment in reality. Some researchers investigated the effect of emotion on cooperation behavior and claimed that emotions could be spread in a PGG. Szolnoki et al. [32, 33] studied a pioneering work about emotion and claimed that a player shows envy to a more successful opponent and sympathy to a less successful opponent. Quan et al. [34] proposed an emotion-imitating rule for strategy updating and found that it significantly boosts cooperation level in social dilemmas with extortion. Wang et al. [35], Ji et al. [34], and Xie et al. [36] found that the diversity of the emotion dramatically increases the cooperation level and produces a high level of social welfare. These investigations regarded the emotion of players toward their opponents as homogeneous, which is unsuitable for emotional investments because a player’s emotions are also different when facing diverse opponents. Usually, a player displays delight to a cooperative opponent and a negative emotion to an opponent with defective behavior. Moreover, a player’s emotions toward his opponents are also dynamically updated in the evolution of cooperation in PGGs. Regarding the effects of emotion on the investment in realistic situations, a player often invests more in an opponent if he is glad with this opponent while invests less or even nothing if he feels angry with this opponent. Additionally, players in real life have a memory capacity, which also influences the update of the players’ emotions and the decision-making in investments [37–40]. Generally, the memory has a continuous decay with the passage of time, and the current memory is the most impressive. In other words, emotion is a result of the memory-cumulative process. For an incomplete rational person, his investment is most susceptible to rational and emotional factors. Does the emotional investment conflict with the rational investment? How do the rational factors and the emotional factors affect the investments? What is the evolution of cooperation in a heterogeneous investment caused by these factors? Their problems are still mysterious to us.

Motivated by these discussions, we considered the payoff as the rational factor and introduced the effects of heterogeneous investments caused by players’ payoffs and the emotions on the evolution of cooperation in PGGs. Firstly, we introduced a memory length and a memory capacity coefficient to describe the memory-cumulative effect. Then a new emotional correlation between a player to different opponents was proposed according to the memory-cumulative effect. Secondly, an investment formula was proposed based on emotion and the effective payoff. Aiming at describing the contributions of the emotional investment and the rational investment, we used two parameters *α*_*1*_ and *α*_*2*_ to characterize the strength of the emotional investment and the rational investment. At last, we examined the effect of the emotion and payoff on cooperation behavior in a PGG. The results show that there is a mutually reinforcing relationship between emotional and rational investment.

This paper is organized into the following parts. Section 2 introduces the model of heterogeneous investments induced by emotions and payoffs. Section 3 presents the results and discussion. Section 4 draws conclusions.

## Models and methods

**Fig 1:**
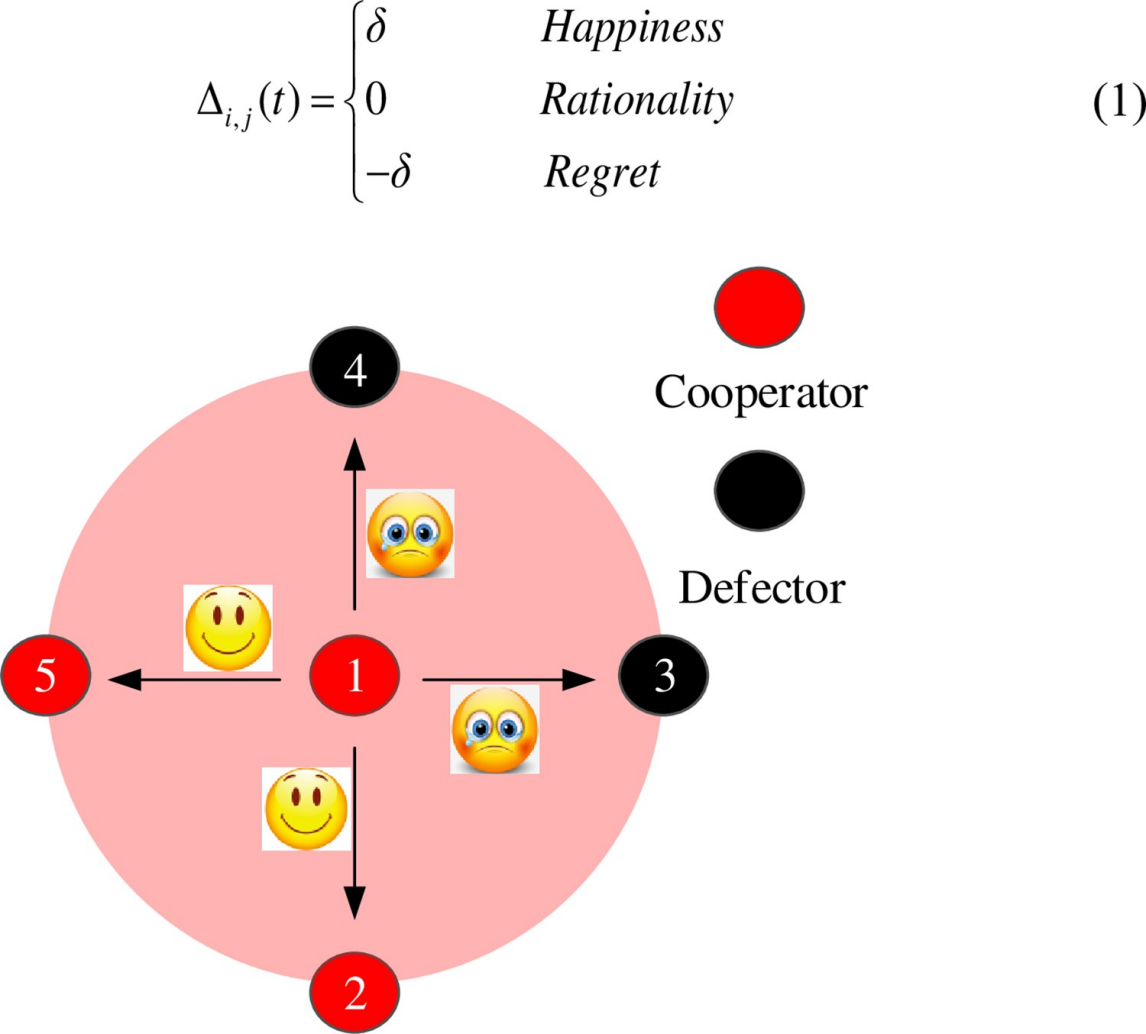
Emotion generation of a player when investing to neighbors with different strategies.

Emotion is an essential part of our investment decision-making. It dynamically changes when facing different players. As shown in Fig 1, a cooperator (C) will be pleased when finding that his investment is contributed to a cooperator and will be regretful if he finds that his investment is contributed to a defector (D). In contrast, a defector will keep rational because he doesn’t contribute his investment to anybody. Thus, the emotion changes in every round in PGG. To quantify the emotional change in every round, we define an emotional unit *δ*. The emotional index of a cooperator increases *δ* when coming across a cooperator neighbor and decreases *δ* when facing a defector neighbor. While a defector remains unchanged in his emotional index. Thus, the emotional change in every round could be expressed as follows,

$$\Delta_{i,j}(t)=\{\begin{array}{l} \delta \;Happiness\\ 0\;Rationality\\ -\delta \;Regret \end{array}$$ (1)

At the beginning of this game (*t* = 1), all players keep a neutral attitude and accumulate their emotions according to their neighbors’ behaviors. Furthermore, this cumulative emotion is affected by memory capacity. Regularly, the current emotion is the most impressive, and the historical emotion gradually declines with time. Here, we consider a memory length of *m* and a memory capacity coefficient *ε*. Thus, the emotional index of player *i* on player j at step *t* could be expressed as,

$$E_{i,j}(t+1)=\{\begin{array}{l} \sum_{l=1}^{t}\varepsilon^{l-1}\Delta_{i,j}(t-l+1)\;t<m\\ \sum_{l=1}^{m}\varepsilon^{l-1}\Delta_{i,j}(t-l+1)\;t\geq m \end{array}$$ (2)

Where, *E*_i,j_ is the emotional index of player *i* on player *j*, *m* the memory length, *ε* the memory capacity coefficient, 0<*ε* ≤ 1. Once the emotional index of player *i* on player *j* reaches an extremely high or low level, the cooperation or defection behavior will have little impact on the index. Thus, a upper and lower bounds of the emotional index are set,

$$E_{i,j}(t+1)=\{\begin{array}{l} e\;E_{i,j}(t+1)>e\\ E_{i,j}(t+1)\;-e\leq E_{i,j}(t+1)\leq e\\ -e\;E_{i,j}(t+1)<-e \end{array}$$ (3)

Where, *e* is a threshold value.

The total emotion of player *i* can express as,

$$E_{i}(t+1)=\sum_{j\in \Omega_{i}\cap j\neq i}E_{i,j}(t+1)$$ (4)

An investor allocates his capital to neighbors according to his mood and historical payoff. Usually, an investor contributes more to a neighbor who brings more payoff to him or pleases him. Thus, a statistic model is employed to describe the investment rules as follows,

$$I_{i,j}(t+1)=\frac{e^{\alpha_{1}E_{i,j}(t)+\alpha_{2}{\tilde{u}}_{i,j}(t)}}{\sum_{k\in \Omega_{i}}e^{\alpha_{1}E_{i,k}(t)+\alpha_{2}{\tilde{u}}_{i,k}(t)}}$$ (5)

Where, *I*_*i*,*j*_(*t*+1) is the investment of player *i* to player *j* at time *t*+1, and this investment is 0.2 at the initial time. *Ω*_*i*_ the group centered on player *i*. *α*_*1*_ is an emotional weight, and *α*_*2*_ is a rational weight, *α*_*1*_, *α*_*2*_ >0. ${\tilde{u}}_{i,j}(t)$ is the historical payoff and is also affected by the memory capacity, as follows,

$${\tilde{u}}_{i,j}(t)=\{\begin{array}{l} \sum_{l=1}^{t}\varepsilon^{l-1}u_{i,j}(t-l+1)\;t<m\\ \sum_{l=1}^{m}\varepsilon^{l-1}u_{i,j}(t-l+1)\;t\geq m \end{array}$$ (6)

Where, *u*_*i*,*j*_(*t*) is the payoff of player *i* obtained from a group centered on player *j*, and can be calculated as,

$$u_{i,j}(t)=\frac{r}{G}\sum_{k\in \Omega_{j}}I_{k,j}(t)•s_{k}-I_{i,j}(t)•s_{i}$$ (7)

Where, *r* is the multiplication ratio, and *G* is the number of players in a group centered on player *j*.

The total payoff of player *i* at time *t* can be calculated from his groups as follows,

$$U_{i}(t)=\sum_{j\in \Omega_{i}}u_{i,j}(t)$$ (8)

All players update their strategies synchronously by means of the Fermi rule [26]. Player *i* randomly chooses one of his neighbors *j*, and adopts the strategy of player *j* with a probability,

$$W(s_{i}\leftarrow s_{j})=\frac{1}{1+e^{(U_{i}-U_{j})/K}}$$ (9)

Where, *s*_*i*_ is the state value which is 0 for a defector and 1 for a cooperator. *K* is the intensity of noise and is usually set to 0.1, as reported by Zhang et al. [26].

**Fig 2:**
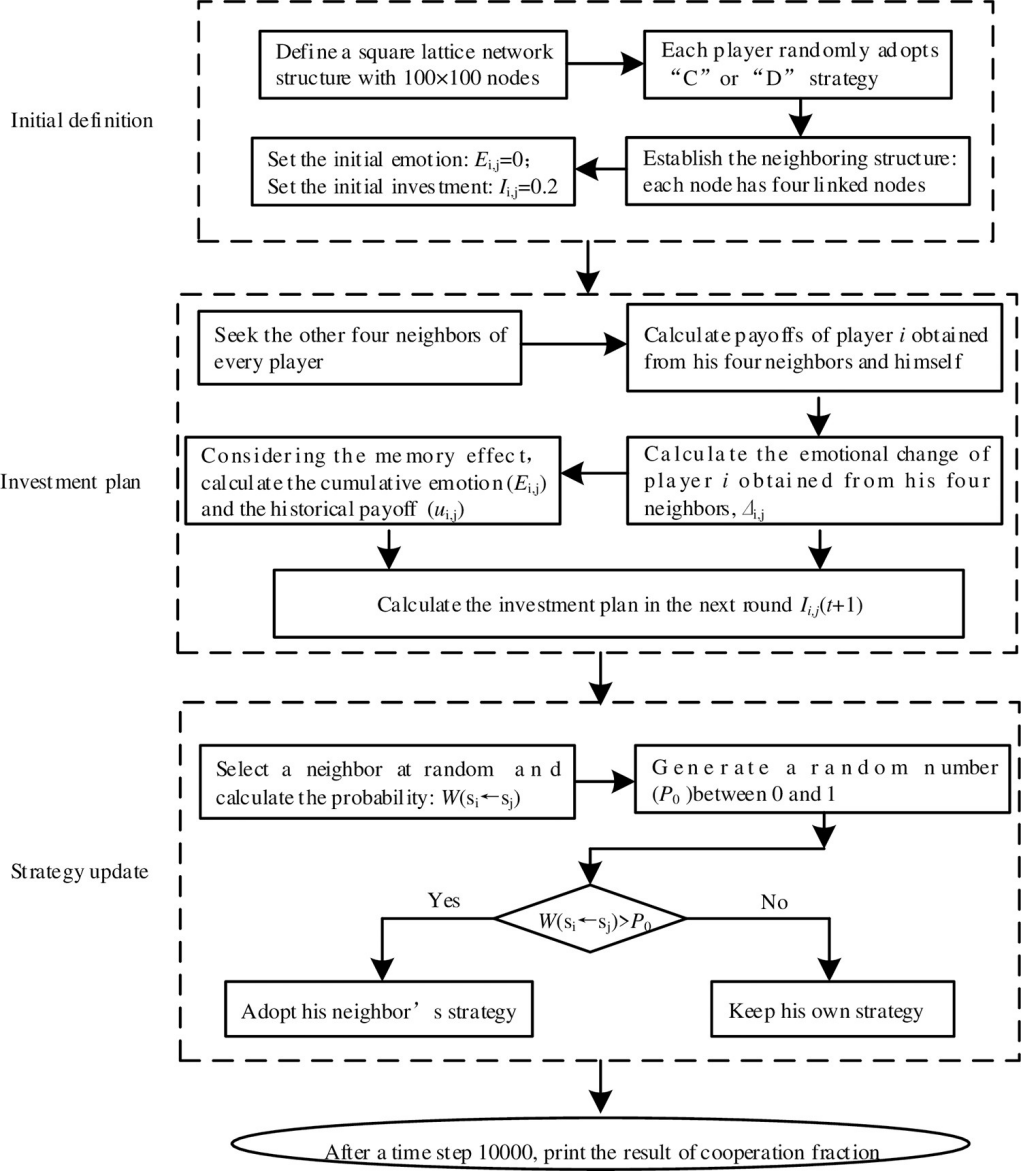
Simulation flow chart.

The paper considers a square lattice network structure with periodic boundary conditions. Matlab software is adopted to simulate the evolution of this game, and its calculation flow chart is shown in Fig 2. A square lattice network structure with 100×100 players is defined. Each player chooses to be a defector or a cooperator with the same probability. In this game, every player has four linked neighbors. We also designed a two-dimensional plane to describe the location of each player and the linked structure. For player *i*, its coordinate value in this plan is labeled as (*x*, *y*), and its four neighbors’ coordinate values are (*x+1*, *y*), (*x-1*, *y*), (*x*, *y+1*), (*x*, *y-1*). Initially, each player has the same emotion value of 0 and the same investment value of 0.2 to his neighbors. The payoff of player *i* obtained from his neighbors is the sum of the total investments to his neighbors. The emotional change of player *i* to his neighbors in this round could also be recorded according to the emotion rules in Eq (1). Considering the memory length and the memory decline effect, the emotional index and the historical payoff can be calculated by respectively following Eq (2) and Eq (6). Thus, a new investment plan in the next round is generated by Eq (5). Subsequently, all players update their strategies according to the Fermi rule in Eq (9), and re-enter the next round of this game by obeying the newly generated investment plan and the new strategies. After a sufficiently long time (1×10^4^), the cooperation frequency tends to be stable, and this simulation can be stopped. To analyze the cooperation level, we average the results of the cooperation fraction over 20 independent Monte Carlo simulations.

## Results and discussions

**Fig 3:**
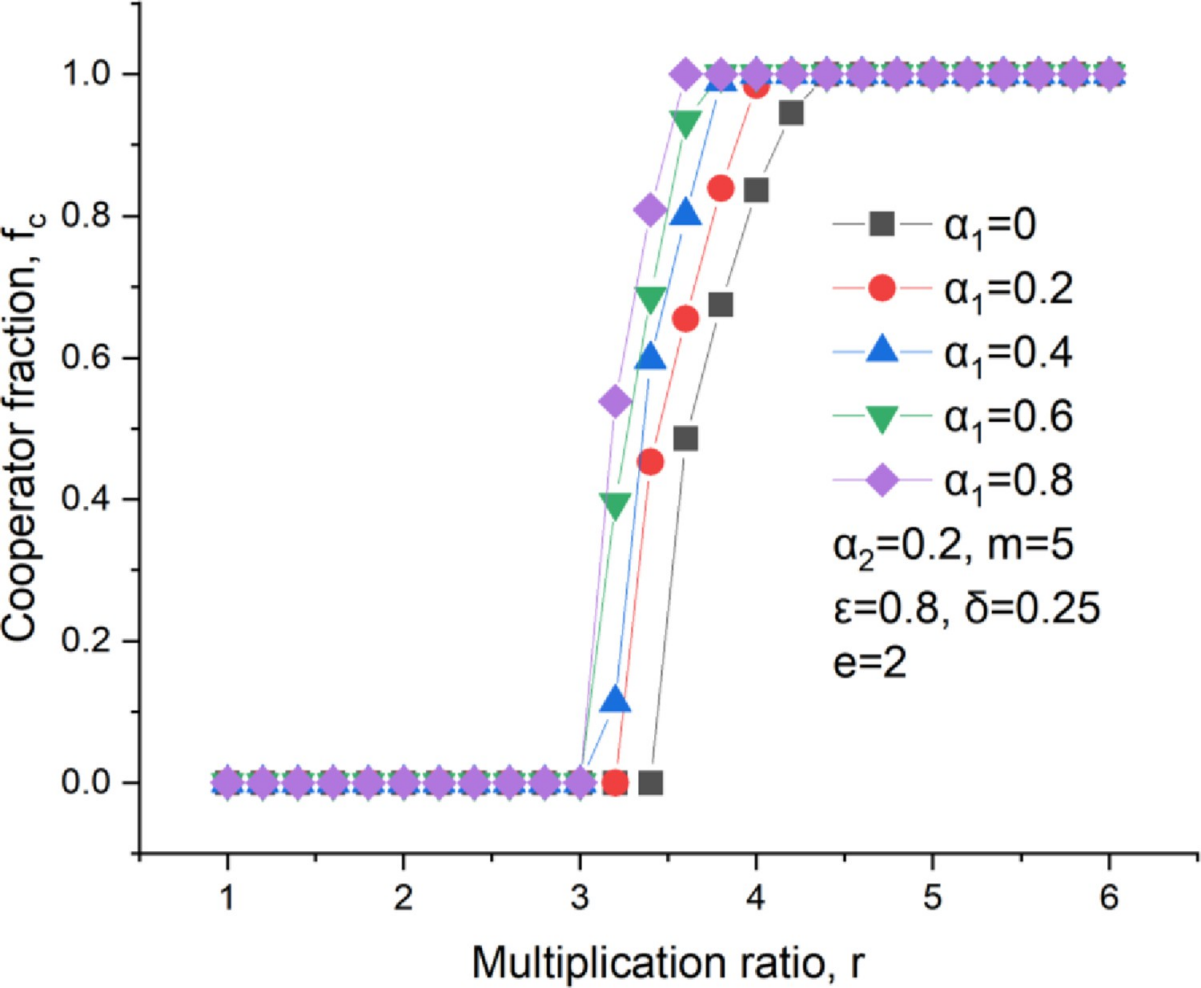
Cooperators level versus the multiplication ratio, *δ* = 0.25, *ε* = 0.8, m = 5, *α*_2_ = 0.2.

Cooperators cannot survive at a small multiplication ratio unless the ratio turns critical, as shown in Fig 3. Once the multiplication ratio exceeds this critical one, the cooperator level begins to increase until to an entire cooperation state. It could be found in Fig 3 that the cooperator fraction stays at 0 when *r* ≤ 3.5, *α*_*1*_ = 0, and that this fraction continuously increases till 1 when increasing the multiplication ratio at *r* ≥ 3.5, *α*_1_ = 0. Comparing the different curves in Fig 3, it could be found that the curve moves to the left with the climbing emotional weight, which means that the emotion results in improving the cooperation level in PGGs.

**Fig 4:**
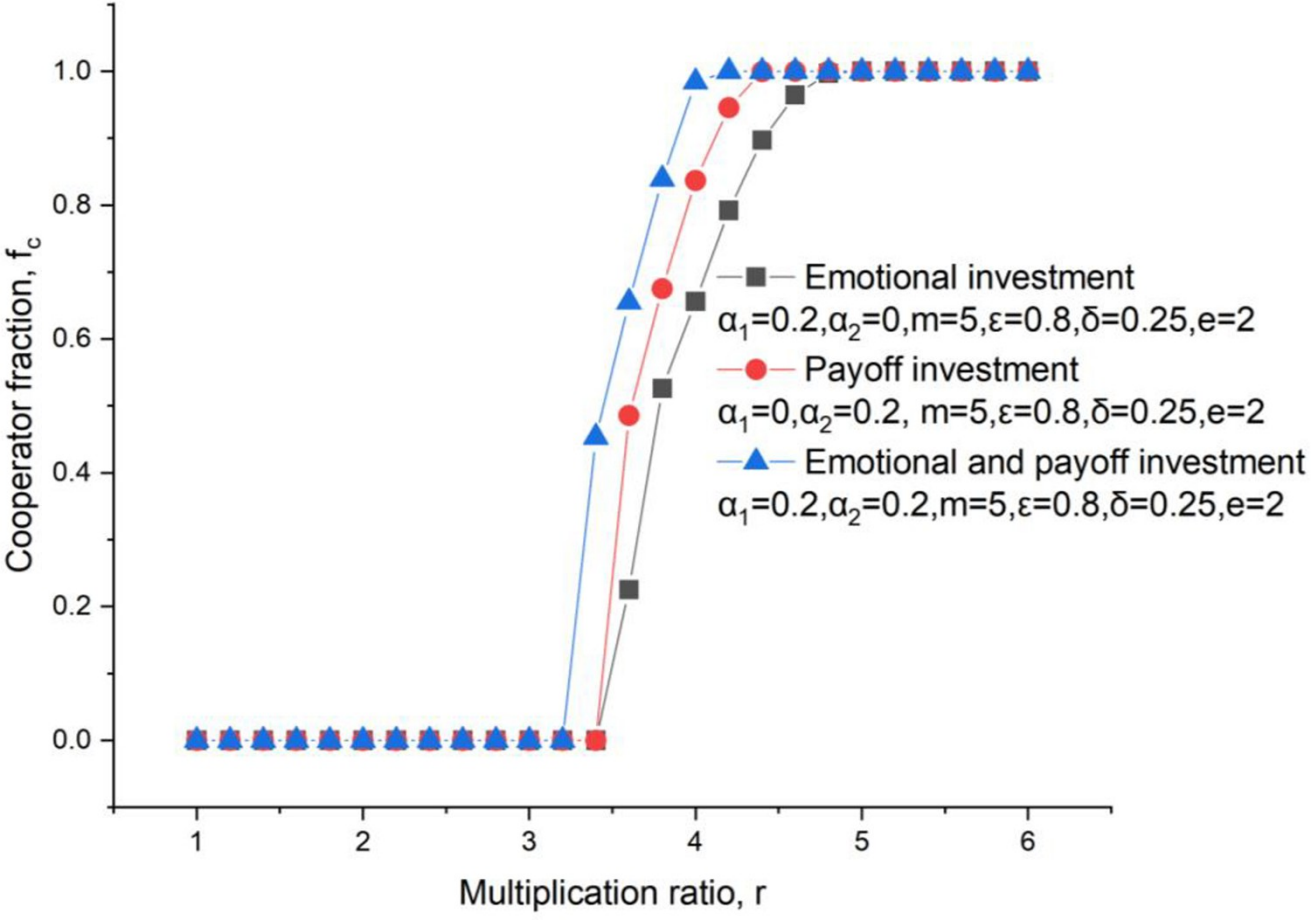
Cooperators level under conditions of the emotional investment, payoff investment, and mixed investment, *δ* = 0.25, *ε* = 0.8, m = 5, *α*_2_ = 0.2, *e* = 2.

Either the emotion or the payoff can be used to help the investment, but there are differences when taking account of this mixed investment. As shown in Fig 4, the critical multiplication ratio appears earliest when using the mixed investment. That’s means an incomplete rational investment is better for promoting the cooperator fraction comparing with the rational investment or the emotional investment.

**Fig 5:**
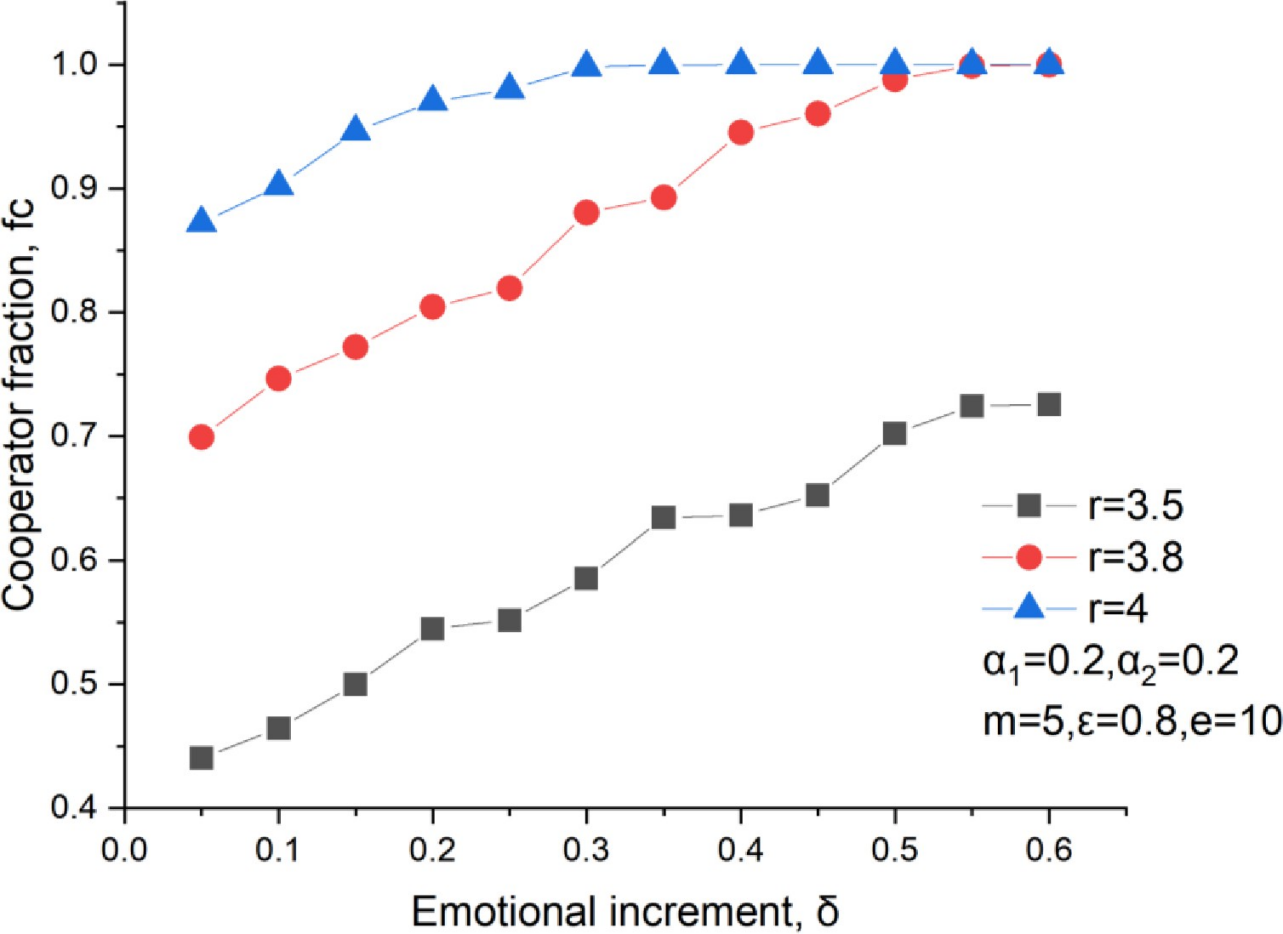
Cooperators level versus the emotional increment, *δ* = 0.25, *ε* = 0.8, m = 5, *α*_1_ = 0.2.

The emotional increment in PGGs could significantly improve the player’s enthusiasm for the investments. Fig 5 shows the cooperator fraction versus the emotional increment at different multiplication ratios. It could be found that the cooperator fraction rises at a large emotional increment, as shown in Fig 5. For a huge emotional increment, a cooperator is more welcomed and gets more investments from his neighbors. At the same time, a defector is disgusted by everyone and gets less investment or even none from his neighbors. Thus, a large emotional increment could form a reward and punishment effect on the players with different behaviors. Form the perspective of this, the emotion plays an important role in an orientation-driven migration in the investment. Just like the location migration proposed by Xiao et al. [41] and Li et al. [42], a large emotional increment can efficiently block an invasion of defective neighbors by contributing little investment or even nothing to defective neighbors. If a cooperator is in a bad situation, such as defective neighbors are in overwhelming major, this cooperator will stop his investment in the next round by changing his cooperative behavior. That’s to say, the emotion helps cooperators protect their interests by escaping away from their defective neighbors or even by giving up his investment in a bad situation. Compared with the location migration, it has no migration cost and has no need to determine where to move.

**Fig 6:**
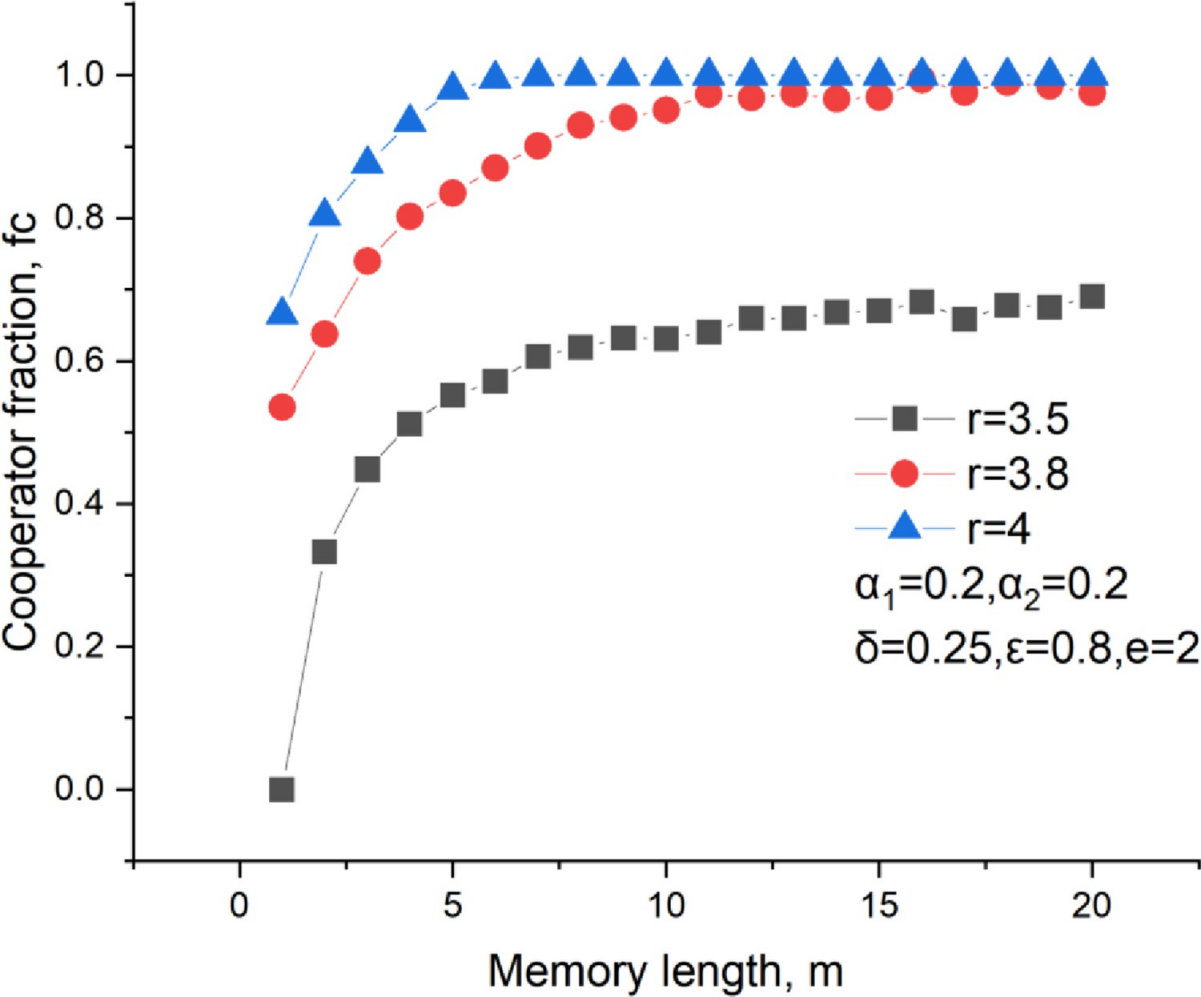
Cooperators level versus the memory length, *δ* = 0.25, *ε* = 0.8, *α*_1_ = 0.2, *α*_2_ = 0.2.

The memory length also affects the cooperation level, as shown in Fig 6. It could be found that a larger memory length brings a higher cooperator fraction, which is also consistent with that in reality. As we know, everyone can remember the historical behaviors of his neighbors and the historical payoffs obtained from them. If one always keeps cooperative, he will get more trust from others and also more investments. However, if one constantly acts defectively, he will be repelled by others and acquired fewer investments. Similarly, players like to invest more into a neighbor who rewards a larger payoff. However, the memory length is not suggested as larger as better. The cooperation fraction grows slowly with an increase in the memory length when it is long enough. It means that a proper memory length should be advised to store the historical behaviors and payoffs for optimal investment. Moreover, we do not suggest an overlong memory length in true life because it is a waste of storage space and cannot facilitate cooperation much.

**Fig 7:**
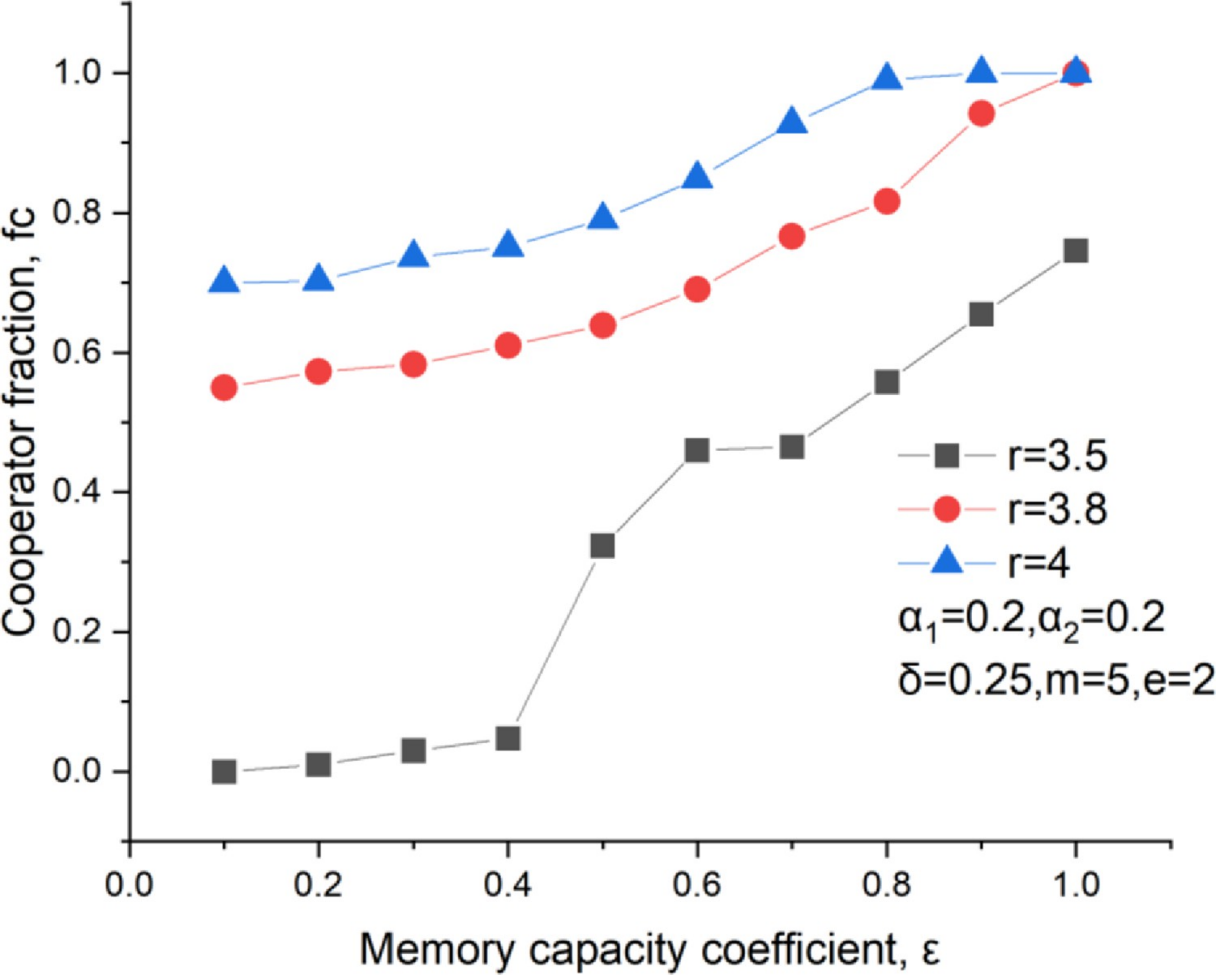
Cooperators level versus the memory coefficient, *δ* = 0.25, m = 5, *α*_1_ = 0.2, *α*_2_ = 0.2.

The memory capacity coefficient also affects the play’s investment. It could be found that a significant memory capacity coefficient results in a high cooperator fraction, as shown in Fig 7. That is to say, poor memory capacity reduces the cooperation level. A poor memory capacity makes players gradually forget the historical behavior and the historical payoff. So, a player who used to take a defection strategy also has many chances to get more investments if he changes his mind to be a new cooperator. This is good news for those defectors to start with a clean SLATE, but it also brings small living space for some opportunists to choose to be defectors with a minor frequency who seek the maximum personal interest. Especially at a small multiplication ratio *r* = 3.5 and a small memory capacity coefficient *ε*≤ 0.4, the cooperator fraction stays below 0.1, as shown in Fig 7. It denotes that most people are more likely to be an opportunist rather than a cooperator.

**Fig 8:**
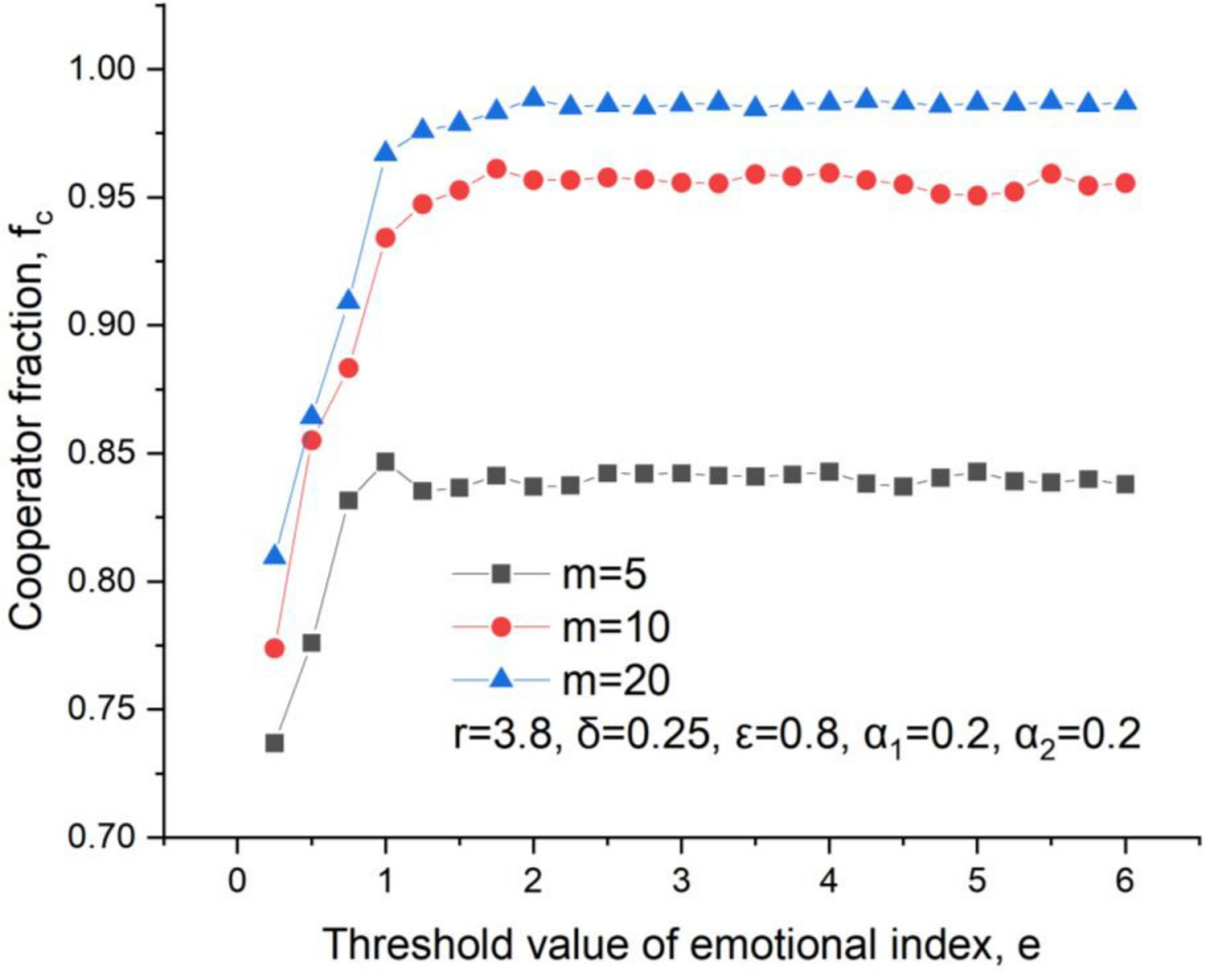
Cooperators level versus threshold value, *δ* = 0.25, *ε* = 0.8, m = 5, *α*_2_ = 0.2.

The threshold value of the emotional index also affects the cooperator fraction, as shown in Fig 8. It could be found the cooperator fraction continuously increases until to a stable one with an ascending threshold value. As we know, once an emotional index overflows the threshold value, a senior cooperator will not get more investment and a senior defector will not get more punishment. That’s to say, a small threshold value is not suggested because it limits the growth of the cooperator fraction.

**Fig 9:**
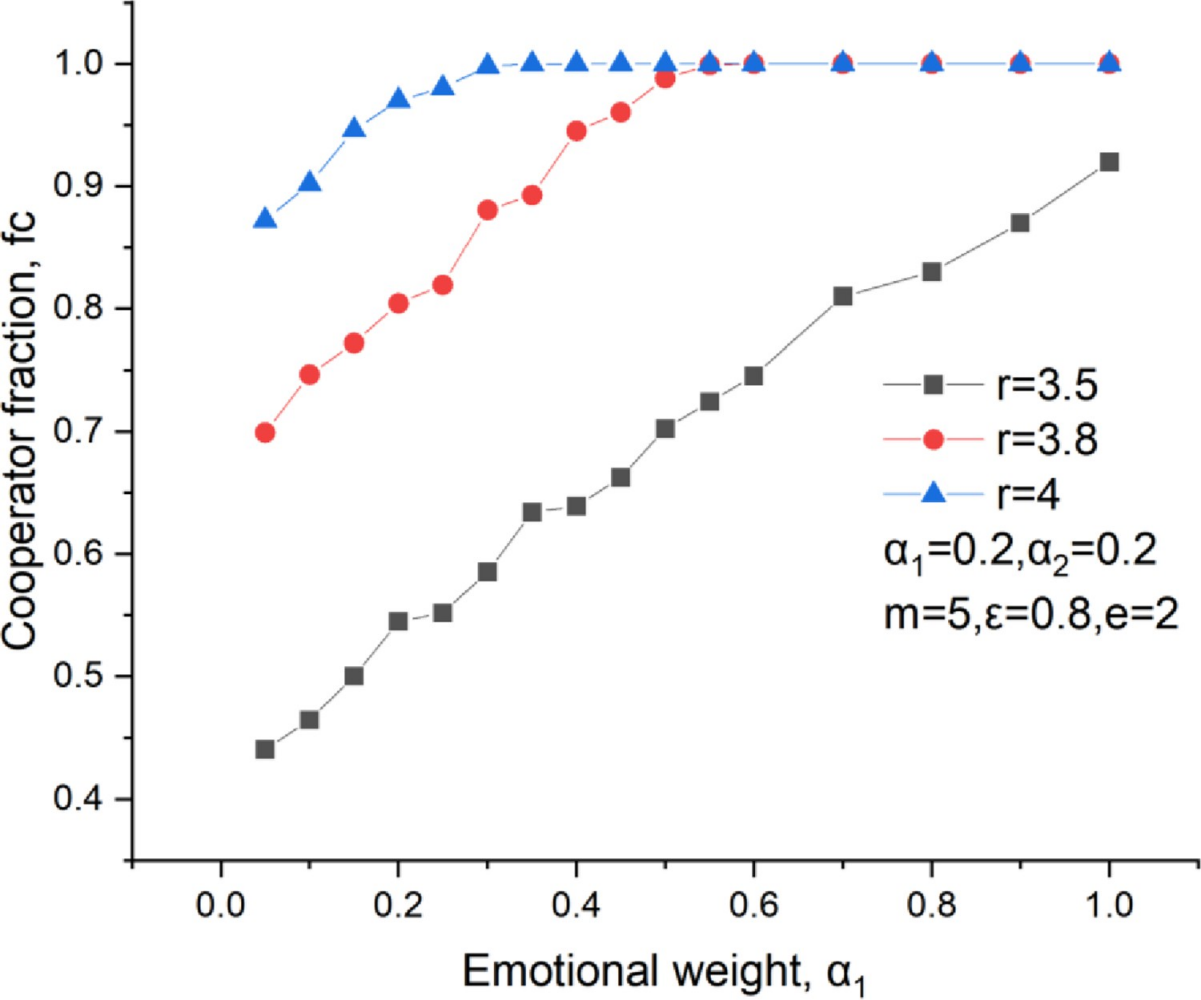
Cooperators level versus the emotional weight, *δ* = 0.25, *ε* = 0.8, m = 5, *α*_2_ = 0.2.

**Fig 10:**
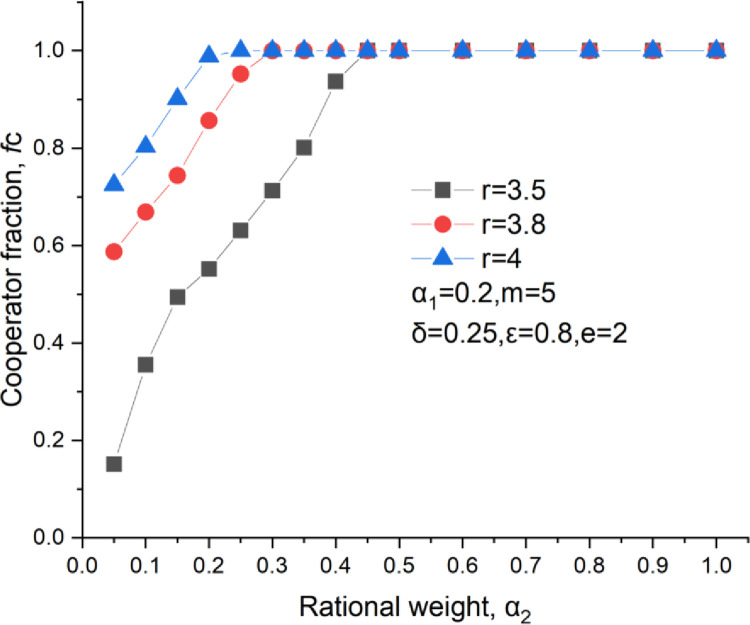
Cooperators level versus the rational weight, *δ* = 0.25, *ε* = 0.8, m = 5, *α*_1_ = 0.2.

**Fig 11:**
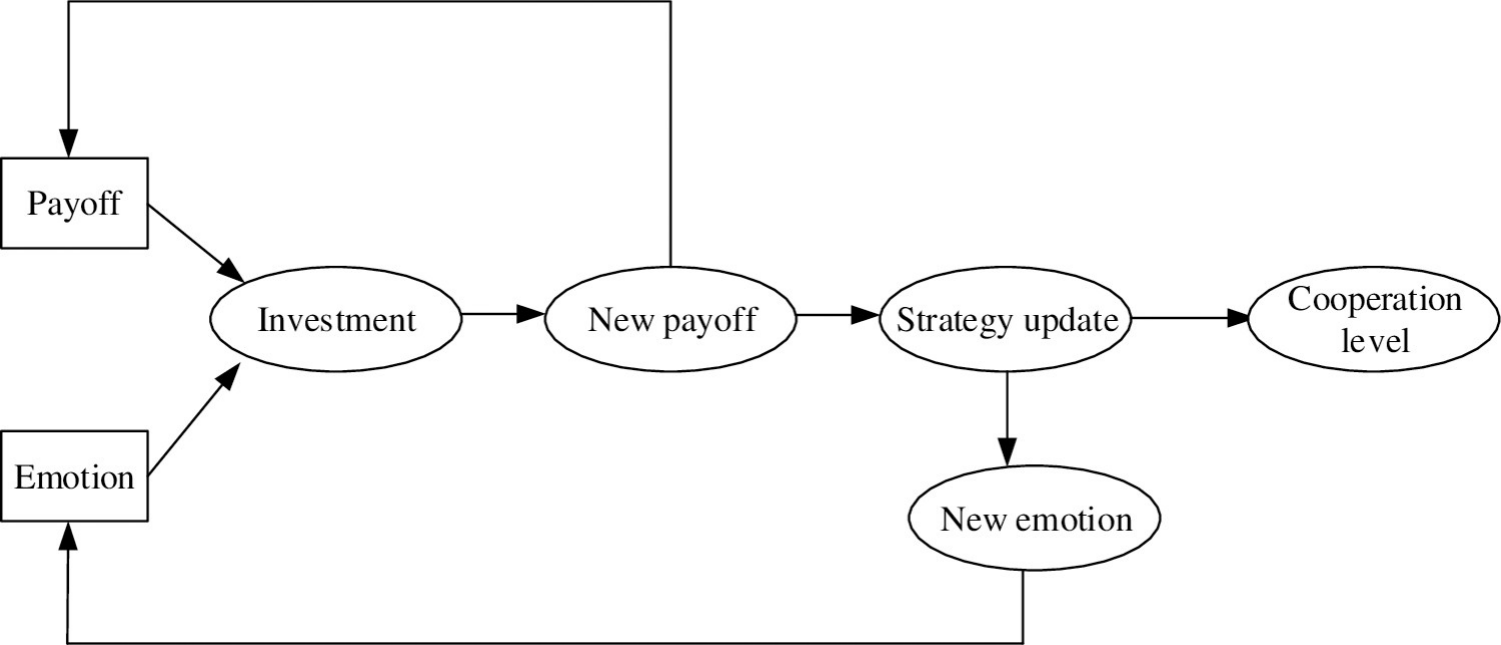
Feedback links by employing the emotional and rational investments.

Fig 9 shows the curve of the cooperator fraction versus the emotional weight. We find that an increase in emotional weight with the cooperation level up, proving that emotion significantly rises the cooperation level. Fig 10 illustrates the curve of the cooperator fraction versus the rational weight. We learn that the cooperator fraction increases with the growth of the rational weight. Thus, both emotional and rational investments can increase the cooperation level. There are two positive feedback links for these two investment methods, as shown in Fig 11. They are the rational and the emotional feedback links. In terms of the rational feedback link, a cooperator usually invests more in a neighbor who may give an enormous payoff in the next round, which results in a further investment increase to this neighbor. Regarding emotional feedback, a cooperator tends to invest more in one of his neighbors who behaves cooperatively frequently. This will bring a higher payoff to this cooperator neighbor, which further encourages him to maintain cooperation behavior in the subsequent rounds.

There are some differences and connections between these two feedback links. The rational investment mainly focuses on the group income because a player decides his investment according to the historical payoff from the group centered by one neighbor. If the centered player is a defector and his group income is high, people are also willing to invest in him. That is to say, the rational investment has a rough tuning but immediate effect in driving the investments into the cooperator cluster with a high group income. In contrast, emotional investment has a highly targeted effect. One would invest less or none in his neighbors even though these defectors’ group has a high income. Thus, the emotional investment has a fine-tuning effect in driving the investment to a cooperator. Also, these two investment methods enhance mutually. Emotional investment promotes rational investment. One invests in the neighbor who makes him happiest and gets a large payoff in the next round. This further promotes rational investment because he will invest more in this neighbor once he receives a significant payoff from this neighbor.

**Fig 12:**
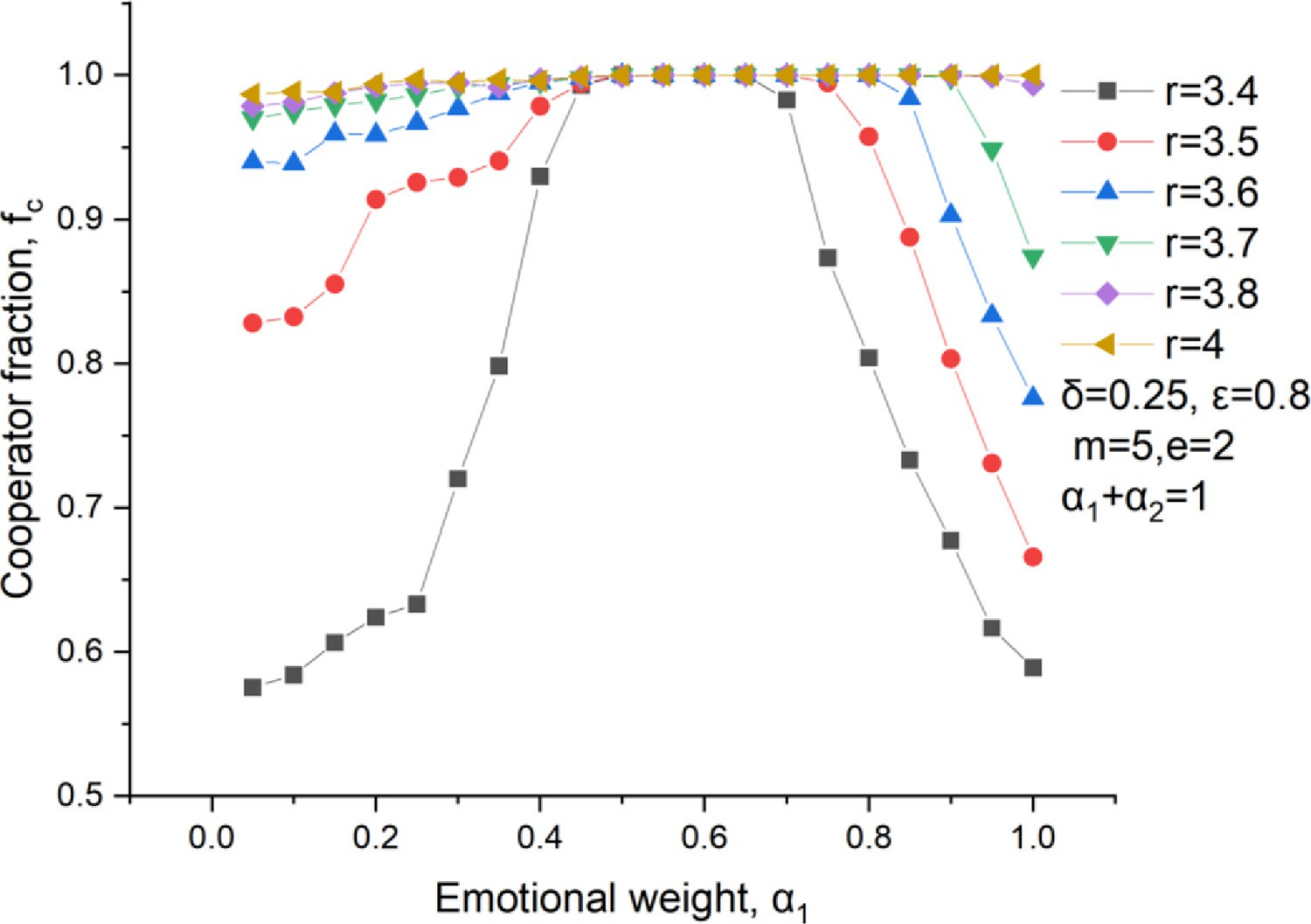
Cooperators level versus the emotional weight at *α*_1_+*α*_2_ = 1, *δ* = 0.25, *ε* = 0.8, m = 5.

If there is a constraint between the rational weight and the emotional weight, such as, *α*_*1*_*+α*_*2*_ = 1, the effect of the emotional weight on the cooperator fraction is more complex as shown in Fig 12. We defined that players tend to be emotional investors when *α*_*1*_ >0.5 and that players are inclined to choose rational investment when *α*_*1*_ ≤ 0.5. For a large multiplication ratio, *r* ≥ 3.8, its cooperator fraction is very high and is almost unaffected by the emotional weight because the reward is great for an investor no matter what kinds of his investment ways. For a small multiplication ratio, *r* < 3.8, the cooperator fraction firstly increases to a peak, maintains for a while and then decays with a growing emotional weight. In terms of a player inclining toward rational investment, an increase of the emotional weight helps improving the cooperator fraction. For a player tending to be an emotional investor, an increase of the rational weight also contributes to increase this cooperator fraction. All of these further proves that both the emotional and rational investment are important in real life.

**Fig 13:**
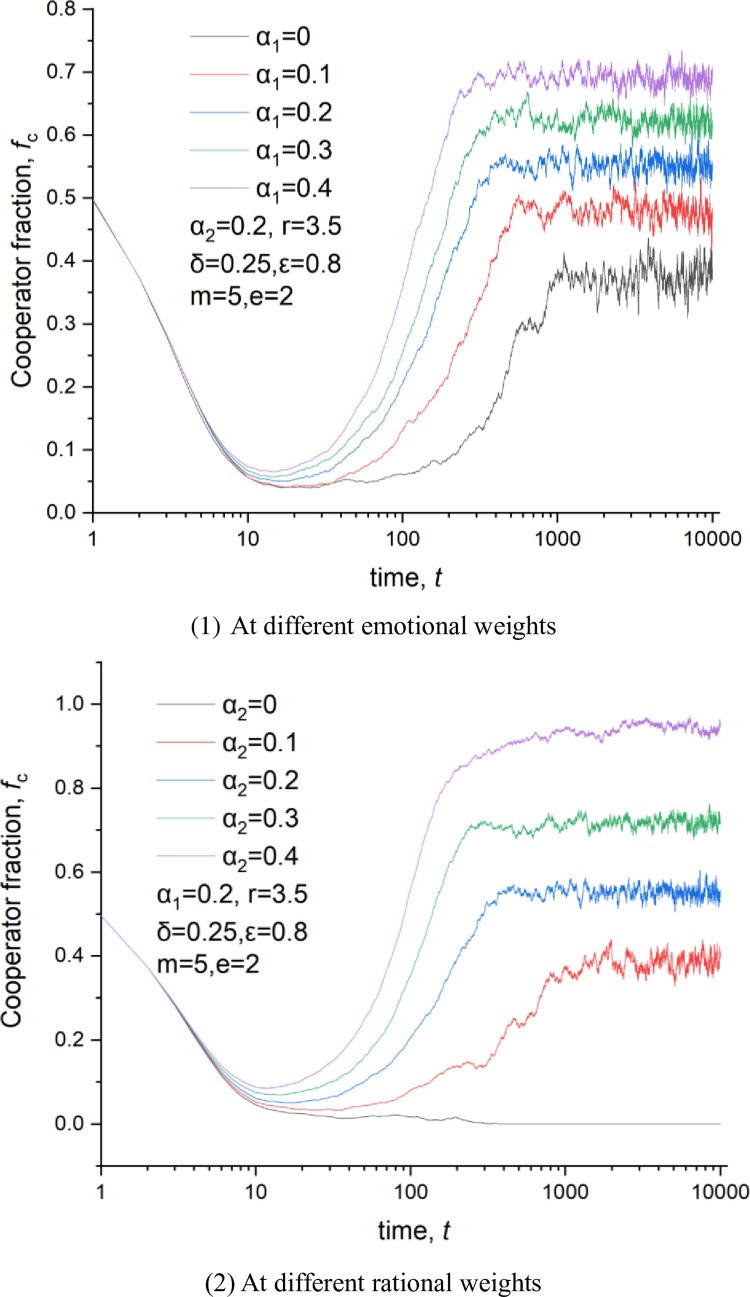
Time series of the fraction of cooperators, *δ* = 0.25, *ε* = 0.8, m = 5, *α*_2_ = 0.2,r = 3.5. (1) At different emotional weights, (2) At different rational weights.

**Fig 14:**
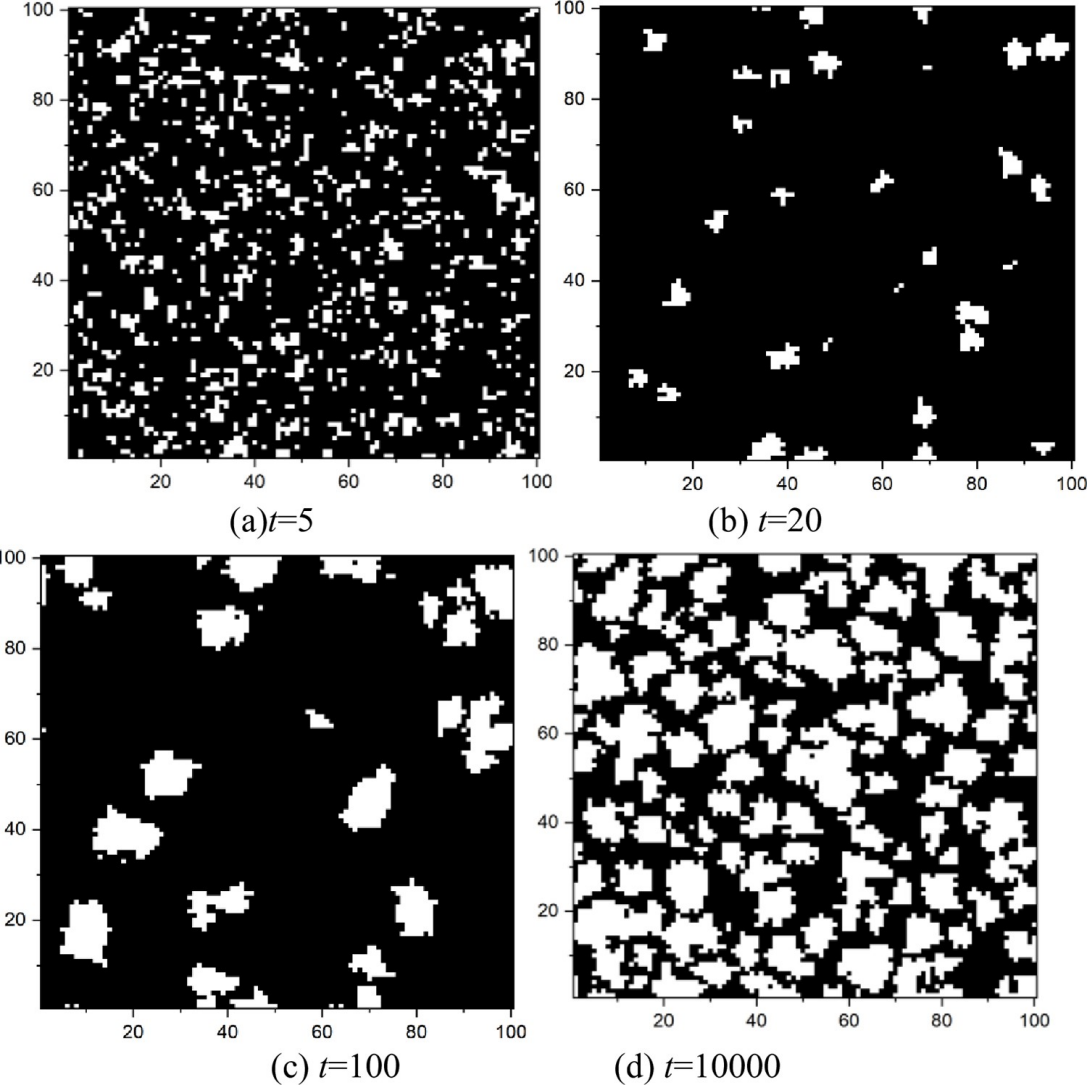
Snapshots of the distributions of cooperators (whiteness) and defectors (blackness) at several representative times, *δ* = 0.25, *ε* = 0.8, m = 5, *α*_2_ = 0.2, *r* = 3.5, *e* = 2. (a)*t* = 5, (b) *t* = 20, (c) *t* = 100, (d) *t* = 10000.

To understand the temporal evolution of the cooperative behavior in PGGs, we offer a time series of the cooperator fraction at different emotional weights and rational weights, as shown in Fig 13. At the beginning, cooperators and defectors are evenly distributed in this network, as shown in Fig 14(A). Thus, defectors could obtain some payoff from their cooperative neighbors even in a grim situation where no one invests in them. So, players prefer to be defectors in the next round. That is why the cooperation fraction will decrease no matter what emotional or rational weights at the beginning of the evolution are, as shown in Fig 13. That also means that the change of the investment could not alter the trend of players for pursuing short-term profits at the early stage because their curves overlap with one another as shown in Fig 13.

The living space for single cooperators is being squeezed over time, and some small cooperator clusters begin to be formed, as shown in Fig 14(B). Therefore, the vast majority of investments are distributed in these cooperator clusters due to the emotional effect and the rational investment. This results in a much higher payoff for players in the cooperator cluster than that in the defector one. The boundary player between the two clusters is more likely to join the cooperator cluster in which he will obtain more. Thus, this cooperator cluster would continuously expand, as shown in Fig 14(C), which could also be proved in Fig 13. The figure displays the rise of the cooperation level with the evolutionary time at 20<*t*<800. Moreover, an increase in the emotional or rational weight could accelerate the expanding velocity of the cooperator clusters because the cooperation fraction increases more quickly under a larger emotional or rational weight at 20<*t*<800, as shown in Fig 13.

When the cooperator cluster expands to a specific scale, the cooperation level reaches a plateau, as shown in Fig 13 at *t*>1000. This state could be called a fully developed network. We can conclude that several opportunists exist in this network from the fluctuation in the cooperation fraction shown in Fig 13. But these opportunists could not stay to be a defector with a long evolutionary time as their behavior would be imitated by others and may result in a new defector cluster with a meager payoff. An increase in the emotional or rational weight could reduce the opportunist number because the fluctuation amplitude decreases at a considerable emotional weight or rational weight, as shown in Fig 13.

**Fig 15:**
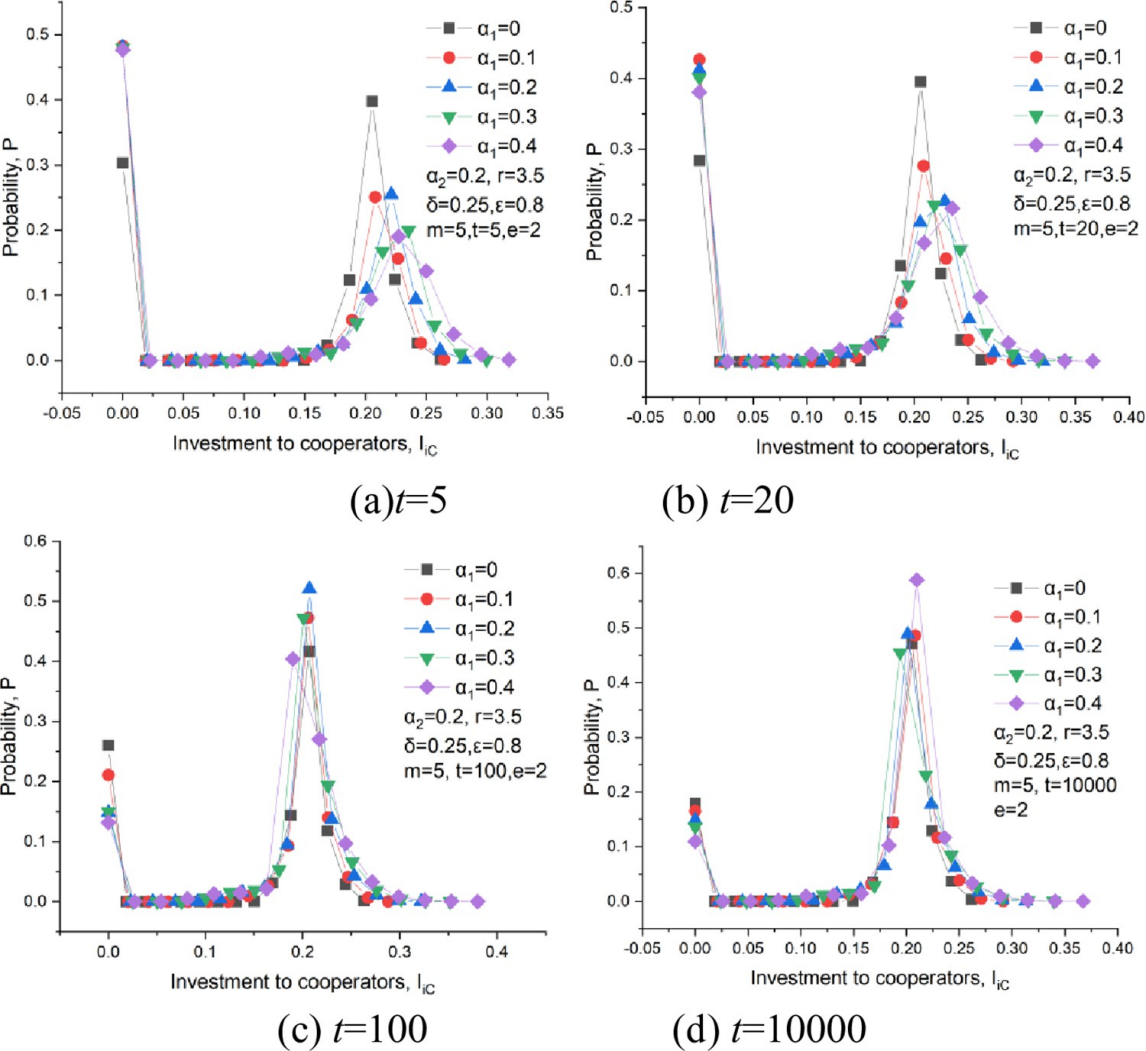
The probability distribution of the investments to cooperators at *δ* = 0.25, *ε* = 0.8, m = 5, *α*_2_ = 0.2, *e* = 2. (a) *t* = 5, (b) *t* = 20, (c) *t* = 100, (d) *t* = 10000.

The investment probability distributions at some representative times are given out, as shown in Figs 12 and 13. *I*_*iC*_ represents the investment to cooperators, and *I*_*iD*_ the investment to defectors. Regarding the investments to cooperators, all curves in Fig 15 are characterized by a bimodal distribution. The first peak appears at *I*_*iC*_ = 0, which means that some defectors surround cooperators.

At an early stage at *t* = 5, cooperator clusters have not been formed yet. The first crest has a significant probability, as shown in Fig 15(A). It suggests that the number of defectors centered by the single cooperator is vast. The first peak probability under a rational investment condition is much lower than that under an emotional condition, which means that the emotion of a cooperator can drive defectors away from him. The second peak probability decreases with an increase in emotional weight, and the curves at *I*_*iC*_>0.23 show a trend of moving to the right. This indicates players with emotions pay more attention to a player with continuous cooperation behavior. Even if the emotion can change the investment at the early stage, the cooperation level still falls with the same trend when adding this emotional effect, as shown in Fig 13 at *t*<10. This further proves that a change of investment cannot change players’ behavior in pursuing short-term profit.

At *t* = 20, the cooperation cluster begins to be formed. The first peak also keeps a high probability, as shown in Fig 15(B), which means defectors still surround many scattered cooperators. For a large investment, *I*_*ic*_>0.22, its probability seems to climb when increasing the emotional weight, which suggests that a collaborative player is much more welcomed by others. This is also beneficial for the expansion of cooperation clusters. For a small investment, 0.1<*I*_*ic*_<0.15, a small probability appears when rising the emotional weight. This explains that the investment of the cooperator clusters to the boundary members is not high during the expanding process of these cooperation clusters. This is very consistent with the phenomenon in true life that trust for a new member entered into a group requires a process for a track record.

For the developing stage at *t* = 100, as shown in Fig 15(C), its probability at the first peak is much lower than that at *t* = 20. It means that the number of scattered cooperators decreases with the evolutionary time and that cooperators try to survive in the form of cooperation clusters. For the developed stage at *t* = 10000, as shown in Fig 15(D), the cooperation clusters stop expanding and keep in a dynamic equilibrium state. The probability at the first peak reaches its minimum and declines with a growth in emotional weight.

**Fig 16:**
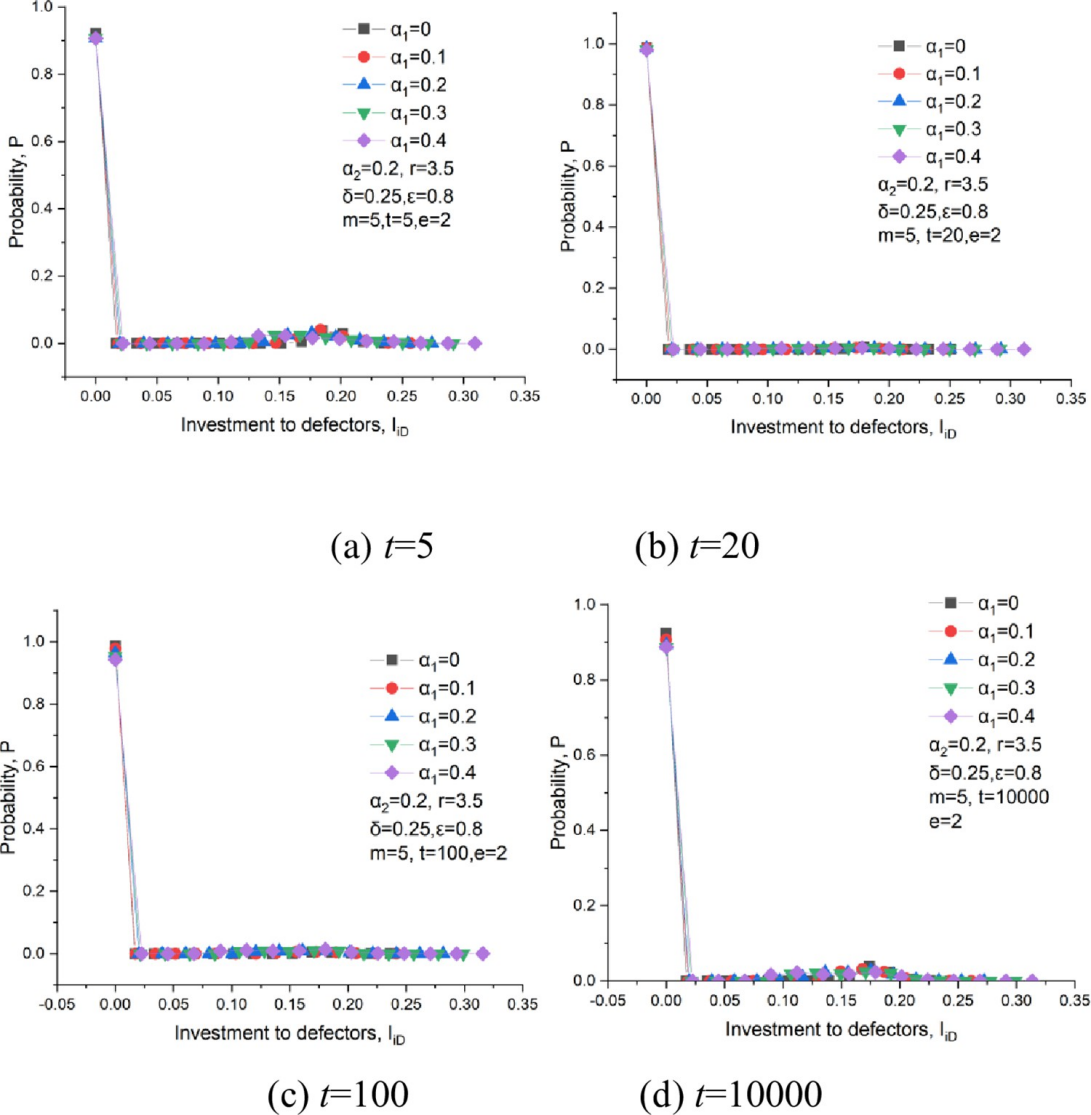
The probability distribution of the investments to defectors at, *δ* = 0.25, *ε* = 0.8, m = 5, *α*_2_ = 0.2, *e* = 2. (a) *t* = 5, (b) *t* = 20, (c) *t* = 100, (d) *t* = 10000.

The investment probability distribution to defectors at some representative times is provided, as shown in Fig 16. The probability at *I*_*iD*_ = 0 is huge and is approximate 1 at different times. This indicates that most players are unwilling to invest in defectors. At *t* = 5, a small probability appears at 0.12<*I*_*iD*_<0.22. This is because the scattered cooperators are surrounded by defectors at the early stage and have to invest in these defectors. At *t* = 10000, a small probability could also be seen at 0.12<*I*_*iD*_<0.22. This is because opportunists existing in this network by frequently switch strategies between a cooperator and a defector.

**Fig 17:**
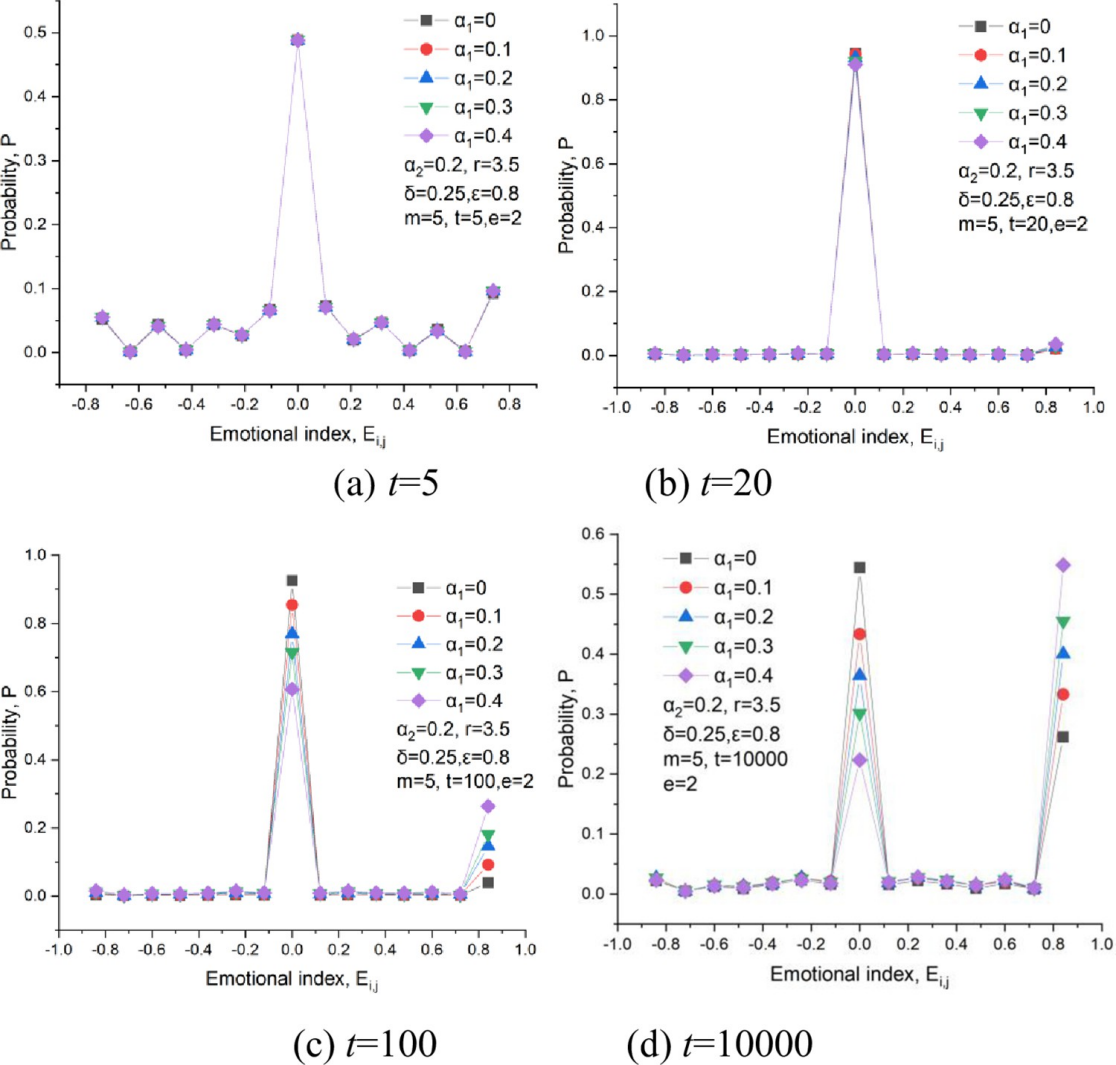
The probability distribution of the emotional index at several representative times, *δ* = 0.25, *ε* = 0.8,m = 5,*α*_2_ = 0.2, *e* = 2. (a) *t* = 5, (b) *t* = 20, (c) *t* = 100, (d) *t* = 10000.

**Fig 18:**
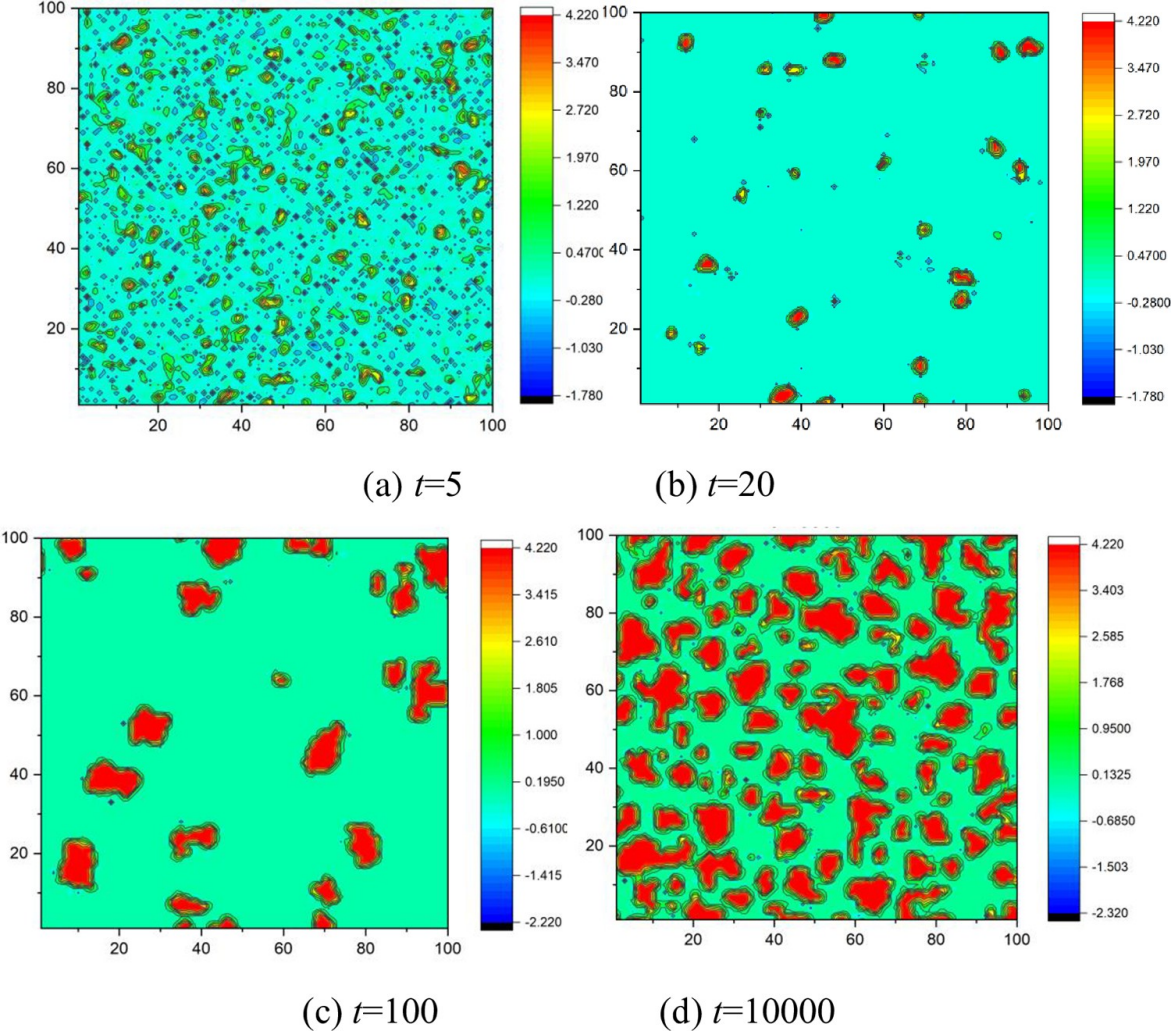
Snapshots of the player’s emotional index at several representative times, *δ* = 0.25, *ε* = 0.8, m = 5, *α*_2_ = 0.2, *α*_1_ = 0.1, r = 3.5, *e* = 2. (a) *t* = 5, (b) *t* = 20, (c) *t* = 100, (d) *t* = 10000.

The probability distribution of the emotional index at some representative times is given out, as shown in Fig 17. Some fluctuations characterize the emotional index, and its peak value reaches at *Eij* = 0 with a large probability of 50%, as shown in Fig 17(A). This is consistent with the distribution of the emotional index in Fig 18. Firstly, half of the players stay rational, and the other half have unstable emotions at the beginning of games. Moreover, the effect of the emotional weight on the emotional distribution is not apparent because the punishment intensity to defectors is not strong enough to stop players from pursuing for the short-term profits.

At *t* = 20, most players stay rational, and a small part has an extensive emotional index of 0.85, which signifies that cooperators begin to show an agglomeration behavior and that some small-scale cooperator clusters begin to form, as shown in Fig 18(B). Compared with the curves in Fig 17(B), the emotional index does not fluctuate because the distribution of cooperators in this network has entered into a well-organized state.

At *t* = 100, the players’ emotion is also very stable, and their curves are characterized by a bimodal distribution. The first peak also appears at *E*_*ij*_ = 0 with a considerable probability, and the second peak at *E*_*ij*_ = 0.85. This could be proved in Fig 18(C). The second peak increases with an increase in emotional weight, which indicates that emotion can promote the growth of the cooperation cluster. At *t* = 10000, the second peak has a high probability, which means most players have a happy mood, as shown in Fig 18(D). Fig 17(D) also demonstrates that the second peak is much higher than the first peak when *α*_*1*_≥0.2, revealing that most players show a pleasing mood in the games.

## Conclusions

The cooperation level induced by the emotional and rational investments in a PGG is investigated. The generation and evolution of players’ emotions are clarified and quantitatively defined by the emotional index. We also examine the effects of the emotional, memory and rational investment parameters on the cooperation level. The emotional increment could be found to significantly improve the players’ enthusiasm and enhance the cooperation level in PGGs. There is no contradiction between emotional investment and rational investment. Regarding the cooperation level, there is a mutually reinforcing relationship between emotional and rational investment. The rational investment mainly focuses on the group’s total income and ignores the strategies of the group members. This investment has a rough-tuning effect on driving the investments into cooperator clusters. At the same time, the emotional investment focuses on the investee’s strategies and has a fine-tuning effect on driving the investment to a single cooperator. A poor memory capacity coefficient allows defectors to change their behaviors. However, it produces several opportunists who frequently switch strategies between a cooperator and a defector to seek the maximum personal interest. A large memory length leads to a high cooperator fraction. However, it is not suggested to be too large because an overlong memory length has little contribution in improving the cooperation level and wastes the storage space. The effects of the coupled investments induced by emotions and payoffs on the cooperation level are consistent with what we discover in real life. We hope our findings can provide more perspectives on understanding the cooperation phenomena in reality.

## Supporting information

S1 Data (XLSX)

S2 Data (XLS)

S3 Data (XLS)
